# Modeling Protein–Protein and Protein–Ligand Interactions by the ClusPro Team in CASP16

**DOI:** 10.1002/prot.70066

**Published:** 2025-10-20

**Authors:** Ryota Ashizawa, Sergei Kotelnikov, Omeir Khan, Stan Xiaogang Li, Ernest Glukhov, Xin Cao, Maria Lazou, Ayse Bekar‐Cesaretli, Derara Hailegeorgis, Veranika Averkava, Yimin Zhu, George Jones, Hao Yu, Dmytro Kalitin, Darya Stepanenko, Kushal Koirala, Taras Patsahan, Dmitri Beglov, Mark Lukin, Diane Joseph‐McCarthy, Carlos Simmerling, Alexander Tropsha, Evangelos Coutsias, Ken A. Dill, Dzmitry Padhorny, Sandor Vajda, Dima Kozakov

**Affiliations:** ^1^ Department of Applied Mathematics and Statistics Stony Brook University Stony Brook New York USA; ^2^ Laufer Center for Physical and Quantitative Biology Stony Brook University Stony Brook New York USA; ^3^ Oden Institute for Computational Engineering and Sciences The University of Texas at Austin Austin Texas USA; ^4^ Institute for Advanced Computational Science Stony Brook University Stony Brook New York USA; ^5^ Department of Chemistry Boston University Boston Massachusetts USA; ^6^ Simons Center for Computational Physical Chemistry New York University New York New York USA; ^7^ Department of Biomedical Engineering Boston University Boston Massachusetts USA; ^8^ Department of Electrical & Computer Engineering Boston University Boston Massachusetts USA; ^9^ Division of Chemical Biology and Medicinal Chemistry, UNC Eshelman School of Pharmacy University of North Carolina at Chapel Hill Chapel Hill North Carolina USA; ^10^ Curriculum in Bioinformatics and Computational Biology University of North Carolina at Chapel Hill Chapel Hill North Carolina USA; ^11^ Yukhnovskii Institute for Condensed Matter Physics National Academy of Sciences of Ukraine Lviv Ukraine; ^12^ Institute of Applied Mathematics and Fundamental Sciences Lviv Polytechnic National University Lviv Ukraine; ^13^ Department of Pharmacological Sciences Stony Brook University Stony Brook New York USA; ^14^ Department of Chemistry Stony Brook University Stony Brook New York USA

## Abstract

In the CASP16 experiment, our team employed hybrid computational strategies to predict both protein–protein and protein–ligand complex structures. For protein–protein docking, we combined physics‐based sampling—using ClusPro FFT docking and molecular dynamics—with AlphaFold (AF)‐based sampling, followed by AF‐based refinement. Our method produced numerous high‐accuracy complex models, including cases where AF alone failed, underscoring the critical role of physics‐based sampling alongside deep learning‐based refinement. For protein–ligand docking, we integrated the ClusPro LigTBM template‐based approach with a machine learning‐based confidence model for rescoring. The method preserves conserved interaction fragments derived from homologous complexes, followed by local resampling using physics‐based sampling and a diffusion model. Our template‐based strategy achieved a mean lDDT‐PLI of 0.69 across 233 targets, which was highly competitive. These results demonstrate that combining physics‐based modeling with AI‐driven refinement can significantly enhance the accuracy of both protein–protein and protein–ligand structure predictions.

## 1 Introduction

Understanding the three‐dimensional structures of protein–protein and protein–ligand complexes is crucial for gaining insights into biological function and for structure‐based drug discovery. Experimental techniques such as X‐ray crystallography, NMR spectroscopy, and cryo‐electron microscopy can determine complex structures, but they are time‐consuming and costly, leaving many known interactions unresolved. Hence, computational prediction of such complexes is important.

The CASP16 experiment in 2024 had prediction categories for both protein assembly and ligand pose prediction. In the assembly category, CASP16 (in conjunction with CAPRI) provided targets of protein–protein complexes of varying difficulty, including antibody–antigen interactions and large multi‐subunit assemblies. In the small‐molecule challenge, participants were given the protein sequence and ligand SMILES for 233 complexes spanning four pharmaceutically relevant targets: human Chymase, Cathepsin G, Autotaxin, and SARS‐CoV‐2 main protease. This setup mimicked a real‐world drug discovery scenario in which computational techniques are used to model follow‐up compounds derived from known chemical matter.

A major breakthrough in protein structure prediction occurred during CASP14 [1] with the introduction of AlphaFold2 (AF2) [2], a deep learning‐based approach that can predict monomeric structures with near‐experimental accuracy. Subsequent studies demonstrated that AF2 and related networks can also directly predict certain multimeric complexes by concatenating sequences and treating them as a single fused input with an artificial chain break [3, 4, 5]. Indeed, by using paired multiple sequence alignments or simply offsetting residue indices, AF2 and RoseTTAFold were able to output docked models in more than 50% of protein–protein test cases. However, end‐to‐end AI methods often fail in cases where co‐evolutionary signals are weak or absent, such as viral proteins or antibody–antigen complexes. In contrast, classical docking approaches, such as those based on the fast Fourier transform (FFT) method [6], can exhaustively sample billions of orientations. They have the potential to find near‐native poses even when learning‐based methods fail [7]. However, docking‐based approaches typically lack accurate modeling of flexibility and are prone to generating high‐ranking false positives [8]. These observations suggest that physics‐based and AI‐driven approaches may play complementary roles in complex structure prediction.

Modeling ligand interactions is also a challenging problem. Previously, our group developed a template‐based small‐molecule docking tool, ClusPro LigTBM [9], which performed well in CASP15 [10] and D3R challenges [11]. Template‐based docking leverages known holo structures: If a target ligand shares a significant substructure with a ligand in a known complex, one can “transplant” that pose to the target. ClusPro LigTBM identifies homologous receptors in the PDB that bind ligands that share a large maximum common substructure (MCS) with the target ligand. The target ligand is then overlaid on the position of the template's MCS atoms to anchor local physics‐based sampling. This approach often outperforms blind docking when suitable templates are available. However, previous iterations of ClusPro LigTBM were limited by the fact that MCS alignments may fail to capture chemically important motifs that are not a contiguous common substructure. Furthermore, selecting the best pose from a docking run remained a challenge. Therefore, we hypothesized that addressing more general templates and ML‐based confidence scoring can further improve the results.

Here, we present our CASP16 results using two pipelines: (1) a protein–protein docking protocol that combines AF predictions with physics‐based docking by the program PIPER [12] implemented in the docking server ClusPro [13] and clustering, followed by AF interface refinement; and (2) a protein–ligand docking protocol that combines SMARTS pattern‐based templates pose prediction with ML‐based confidence scoring. We describe each method and discuss their performance in CASP16. Our findings underscore the notion that integrating physics‐based sampling with ML‐based scoring and refinement can achieve state‐of‐the‐art accuracy in biomolecular structure prediction.

## 2 Methods

### 2.1 Protein–Protein Docking Protocol

**FIGURE 1:**
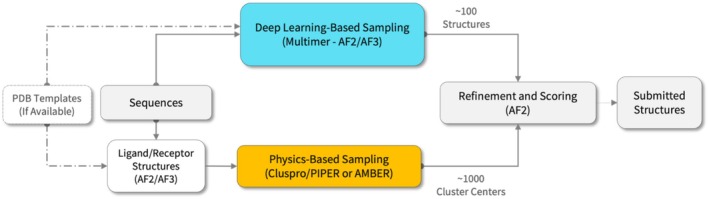
General overview of the methodology employed for modeling of protein multimers. Ligand and receptor denote the smaller and the larger component proteins (see text). For each of the multimeric targets, we generate a large number of multimer poses, using either deep learning (AlphaFold2/3 and their derivatives) or physics‐based (fast Fourier transform‐based docking and molecular dynamics) sampling. The models are pooled together and subjected to refinement and interface‐focused rescoring using the AlphaFold2 template‐based modeling option. Top‐scoring models are submitted as final predictions for each target.

Our protein–protein docking approach consists of two stages (see Figure 1). The goal of the first stage is generating binding pose ensembles by two complementary strategies: (i) physics‐based approaches, including FFT‐docking and molecular dynamics (MD), and (ii) AlphaFold sampling using versions 2.1, 2.3, 3, as well as custom fine‐tuned versions of AF2. The second stage is the local refinement using AlphaFold2.3 and rescoring. The basic idea of the approach follows our previously developed protocol [14], and thus we briefly describe the details below. The major difference from this earlier version of the protocol we used in CASP15 is the scale of sampling we employ for our predictions, with the number of samples submitted to the second rescoring stage increased from dozens to thousands. The human group used the same methodology as the server, but with larger numbers of samples generated and refined/scored as allowed by the extended time limits.

#### 2.1.1 Sampling With AlphaFold

Following previous CASP results, we performed multi‐seed sampling of multimer configurations using available AlphaFold2.1, AlphaFold2.3 and the AlphaFold3 web server [15]. Based on the work by Wallner [16], rerunning AlphaFold with different random seeds changes the subsampling of the Multiple Sequence Alignment input of the model and ultimately results in diversified predictions [16]. Furthermore, for certain classes of targets, such as generic (non‐antibody) protein–peptide targets or the targets involving phosphorylated interface residues, custom fine‐tuned versions of AF2 [17], including Phospho‐Tune [18], were used.

#### 2.1.2 Sampling With FFT

We docked subunits—defined as structures of individual protein chains obtained from unbound crystal structures or AlphaFold‐generated monomer models—using PIPER [12], an FFT‐based docking program developed in our lab and implemented in the ClusPro server [13]. For large multimeric complexes, we docked the largest complex substructures which could be found in PDB or modeled with AF with high confidence. To minimize computational cost by exploiting the characteristics of FFT docking, the smaller subunit was treated as the ligand and the larger subunit as the receptor. An exception was made for antigen–antibody complexes: Due to the use of an asymmetric potential [19], the antibody was always assigned as the receptor. All targets we submitted could be handled in the above manner. Billions of putative docked orientations were generated by combining 70 000 rotations with millions of translations in 3D space (with a translational step of 1.0 Å) for each rotation, and each was scored using a physics‐based energy function that includes van der Waals and electrostatics energies and a pairwise knowledge‐based potential derived by the DARS method [20]. The top 10 000 lowest‐energy poses were clustered by ligand RMSD with a radius of 3 Å. The cluster centers, defined as the structures with the highest number of neighbors within each cluster, were retained for the subsequent refinement/rescoring stage by AF2 (see “AF Refinement and scoring”).

#### 2.1.3 Sampling With MD

For the antibody–peptide systems, where rigid body representation of the peptide is not adequate, our human predictor group used MD as an additional method of physics‐based sampling, with simulations initialized from samples with the highest AlphaFold multimer confidence scores. Antibody–peptide systems were prepared using the tleap program from Amber Tools [21] with a 10 Å cutoff for nonbonded interactions, using particle mesh Ewald (PME) for long‐range electrostatics, and SHAKE applied to constrain bonds involving hydrogen atoms, followed by an eight‐step minimization and equilibration protocol. Firstly, water and hydrogen atom positions were minimized for 100 steps using the steepest descent method, while the rest of the system was restrained with 100 kcal/(mol Å^2^) Cartesian positional restraints. Next, the system was heated to 298 K at a constant volume for 1 ns with 100 kcal/(mol Å^2^) restraints applied to everything except hydrogen and water. The next steps involved 1 ns MD at constant pressure to relax the system density with the same positional restraints. Then, restraint force constants were lowered to 10 kcal/(mol Å^2^) for an additional 1 ns MD at constant pressure. Next, 100 steps of minimization were performed, with restraints applied only to protein backbone atoms using a force constant of 10 kcal/(mol Å^2^). In the next two steps of relaxation, MD used 1 ns at constant pressure, with positional restraints on protein backbone atoms with force constants, respectively, of 10 and 1 kcal/(mol Å^2^). Following equilibration, a 1000‐ns production MD simulation was then carried out under constant pressure and temperature conditions with a 4 fs time step via hydrogen mass repartitioning. Positional restraints with a force constant of 1 kcal/(mol Å^2^) were applied to all antibody atoms except those in the complementarity‐determining regions (CDRs) and the bound peptide. The MD ensemble was formed [22] based on peptide backbone atoms and all heavy atoms, with a minimum inter‐cluster RMSD cutoff of 2 Å.

#### 2.1.4 AF Refinement and Scoring

Each multimer model generated in the sampling stage, whether obtained by physics‐based methods (FFT or MD) or with AF2/3, was used as a template for a subsequent AF2.3 refinement run. In the process, AF2.3, guided by the initial orientation, adjusts side chains and flexible loops at the interface. The refined versions of the samples were evaluated and ranked by a variation of the standard AF2 multimer score, which is the sum of 0.2 PTM (predicted Template Modeling Score) and 0.8 iPTM (interface PTM‐score) computed on interface contacts (residue pairs having atoms within 4.5A) [14].

### 2.2 Ligand Docking Protocol

**FIGURE 2:**
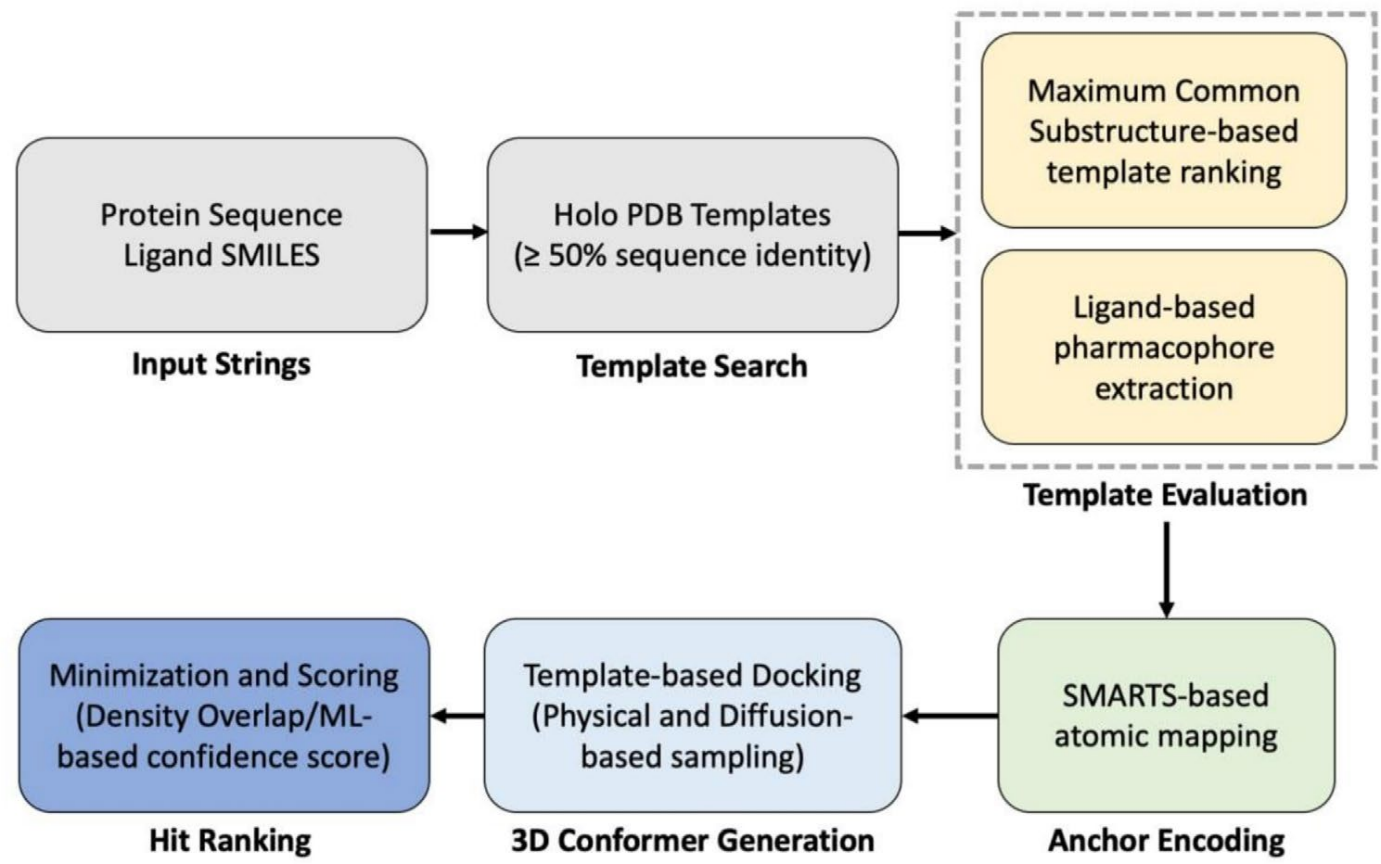
General overview of the methodology employed for modeling of protein–ligand complexes.

Our small‐molecule ligand docking protocol comprises four main stages: template selection and evaluation via SMARTS‐based anchoring, anchor encoding via SMARTS‐based atomic mapping, conformational sampling using ETKDG [23] and a diffusion‐based method, and confidence scoring using a machine learning‐based RMSD predictor (see Figure 2). The approach builds upon and improves prior ClusPro LigTBM methods by integrating flexible pattern‐based anchoring, physically aware sampling, and ML‐based evaluation strategies. Below we provide the key details.

#### 2.2.1 Template Selection via SMARTS Anchoring

For each target receptor, we first identify suitable template structures from the Protein Data Bank (PDB) using MMseqs2 search via the RCSB PDB API. We extract all holo structures of homologous proteins in the PDB that share a ≥ 50% sequence identity with the target receptor. All ligands from these complexes are assembled into a candidate template ensemble. Each template ligand is compared to the target ligand (provided as a SMILES string) using RDKit to compute the MCS, and templates are ranked by the fraction of target atoms covered by the MCS. To guide initial placement, we construct a composite SMARTS pattern that encodes key pharmacophoric motifs conserved across template ligands. To identify key pharmacophoric motifs we construct a ligand‐based pharmacophore from the ensemble of template ligands, and identify interaction sites that are conserved across multiple ligands. First, all template structures are aligned in PyMOL using the align() command. Then, we apply a previously described atomic clustering approach [24] to identify pharmacophore features in crystallized ligands that remain conserved across multiple structures. If moieties included in the MCS overlapped with heavily conserved pharmacophore regions, they were assumed to be important for binding and explicitly encoded in the SMARTS pattern. Unlike strict MCS, the SMARTS formulation allows for chemically analogous but non‐identical substructures to be encoded with a unified pattern. Additionally, SMARTS patterns can represent disconnected substructures, enabling docking to be anchored on conserved motifs that appear in distal regions of a template ligand. Substructures were prioritized for inclusion in a SMARTS pattern if their binding mode was conserved across multiple template ligands. This ensured that SMARTS constraints were typically being applied within a binding hot spot that could form the basis for anchored sampling.

#### 2.2.2 Sampling With ETKDG Conformer Generation

After constructing a SMARTS pattern to map atoms in template ligands to equivalent motifs in the target ligand, we perform constrained conformer generation to sample ligand poses anchored at conserved binding hot spots [24]. For each atomic mapping, up to 1000 conformers are generated using the RDKit implementation of the ETKDG algorithm [23], with SMARTS‐matched atoms fixed to the coordinates of the template ligand. Sampling occurs within the protein pocket, and any conformation which sterically clashes with the receptor (heavy atom overlap < 1.5 Å) is discarded. The remaining conformers are subjected to 500 steps of restrained energy minimization in OpenMM using the AMBER ff14sb force field for the protein, Sage (OpenFF 2.2.0) release of SMIRNOFF for small molecules, and the OBC2 implicit solvent model [25, 26, 27, 28]. Protein backbones are held fixed, whereas ligand and side chains are allowed to move freely. Known coordination geometries (e.g., metal chelation, covalent warheads) are restrained to preserve specific interactions.

#### 2.2.3 Diffusion‐Based Sampling

As alternative to physical sampling, we developed a specialized score‐based diffusion model focused exclusively on torsional degrees of freedom. In the standard paradigm for ligand pose generation, the diffusion process must denoise a state described by translation, rotation, and torsion. Our key insight is that by providing a fixed anchor, a set of atoms with pre‐defined coordinates relative to the receptor, the global translation and rotation of the entire molecule are implicitly determined. This simplifies the generative task significantly, allowing our model to concentrate solely on reconstructing the ligand's conformation by denoising its torsional angles. The model was trained to predict the score of the noise‐perturbed torsion angle distribution, guiding the reverse diffusion process from a random conformational state to a coherent, low‐energy pose similar to DiffDock [29].

For model training and evaluation, we utilized the PDBBind dataset [30]. We adhered to the specific time‐split partitioning and data processing from Stärk et al. [31], which designated roughly 17 000 complexes up to 2018 as the training/validation set. The test set consisted of 363 distinct complexes from 2019, which guarantees no shared ligands with the training data, thus forming a rigorous benchmark for our model's performance.

#### 2.2.4 ML Confidence and Overlap Scoring

To select final predictions, we apply a learned confidence function that predicts the RMSD between a candidate pose and its experimental ground truth. The model's architecture is an SE(3)‐equivariant neural network that operates on the full 3D receptor–ligand geometry as a point cloud, outputting an SE(3)‐invariant scalar which is the estimated RMSD that serves as a direct quality metric. The model is trained on a synthetic dataset derived from PDBbind [30] data. For each sample in the original training set, we generated multiple conformations, resulting in a dataset of predictions with varying RMSD values relative to the ground truth. This synthetic dataset allows the confidence model to learn to estimate the prediction error of the scoring module. The model is then trained to predict the RMSD of a given pose using a Mean Squared Error (MSE) loss. Prior to scoring, we cluster minimized poses by ligand heavy‐atom RMSD using the Butina algorithm as implemented in RDKit with a 0.5 Å cutoff [32]. The lowest energy pose from each cluster is retained. Poses from large clusters that have a high overlap with the template ensembles and a low predicted RMSD score are prioritized as the final submitted predictions.

## 3 Results

### 3.1 Protein–Protein Complex Prediction

**FIGURE 3:**
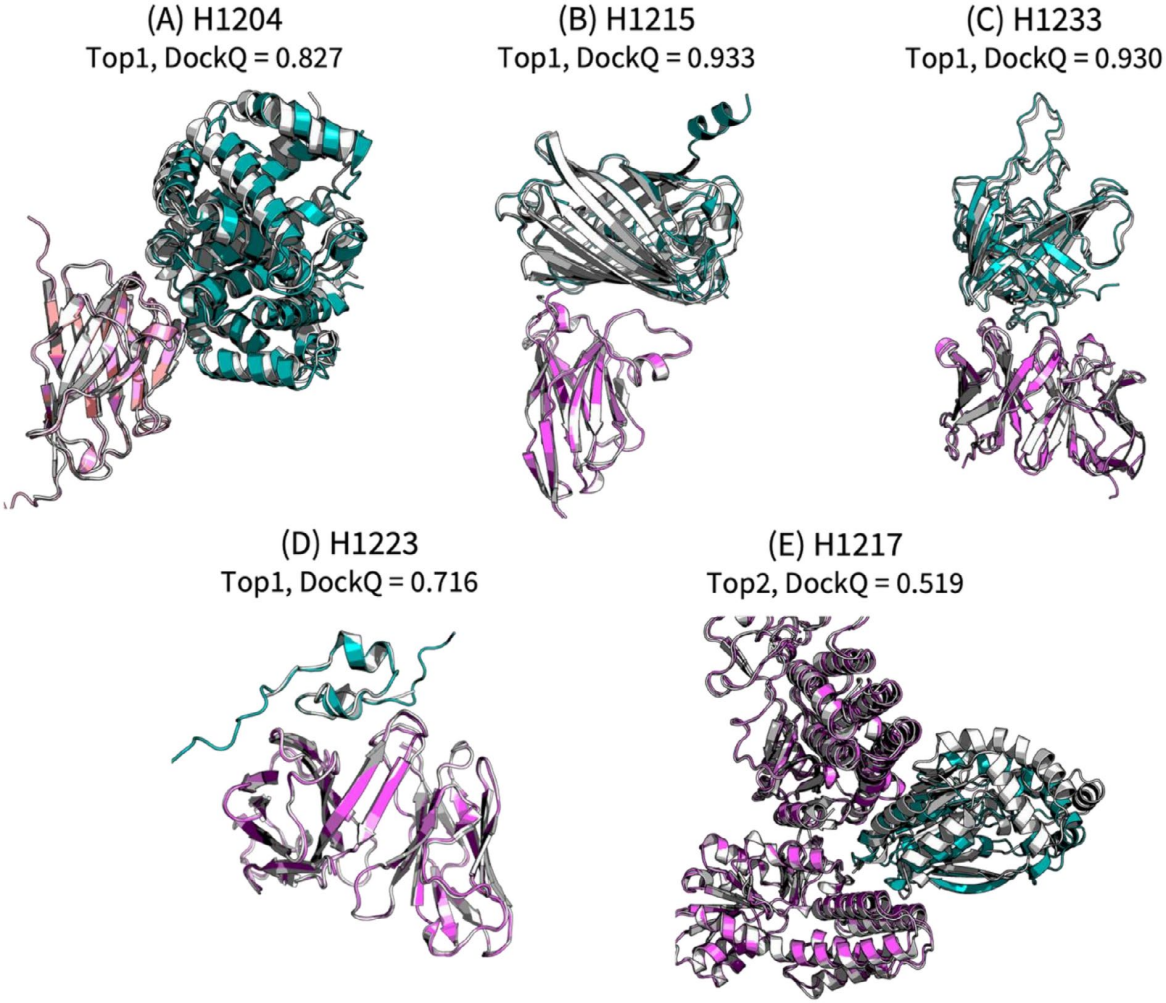
Structural superpositions of predicted and experimental complexes for selected CASP16 targets. Predicted structures are shown in color, and crystal structures are shown in white. To simplify the figure, a protein of interest and its interface partners are displayed. (A–C) Antibody–antigen complexes (antibody in pink; antigens in cyan); (D) antibody–peptide complex (antibody in pink; peptide in cyan); (E) viral protein–translation factor complex (translation factor in pink; viral protein in cyan).

As a CASP16 participating group, we submitted predictions for CASP‐CAPRI multimer targets, and additionally for all antibody–antigen and virus–host targets, which we singled out due to their challenging nature. Combined, this amounted to 35 out of the total 40 CASP16 multimer targets. Our protocol achieved competitive performance among all participants. We focused on antibody–antigen complexes that are generally considered difficult targets [33, 34, 35]. Here we highlight several successful predictions, including some antibody–antigen complexes (H1204, H1215, H1233), an antibody–peptide complex (H1223), and a viral‐translation factor complex (H1217). Figure 3 illustrates these several particularly difficult examples. For the non‐peptidic antibody–antigen complexes (H1204, H1215, and H1233), as well as for the CASP‐CAPRI multimers in general, the submissions were prepared by sampling the poses using ClusPro and AlphaFold2/3, followed by AlphaFold2 refinement and re‐scoring of all samples (see Figure 1). The models with the best DockQ [36] values among our top five submissions for H1204, H1215, and H1233 were each ranked as Top 1 within our submission set, with DockQ [36] scores of 0.827, 0.933, and 0.930, respectively. Note that the DockQ score combines three key metrics from the CAPRI assessment system: fraction of native contacts, ligand RMSD, and interface RMSD. Higher DockQ scores indicate better agreement between the predicted complex and the reference structure. The above scores correspond to CAPRI High‐quality models (DockQ > 0.80). For the viral–translation factor complex (H1217), a similar strategy yielded a model ranked as Top 2, with a DockQ score of 0.519, corresponding to CAPRI Medium quality (DockQ > 0.49).

### 3.2 Antibody–Peptide Targets

In CASP16, several antibody–peptide complexes were included as targets, where accurate modeling required accounting for the inherent flexibility of the peptide—something that rigid docking algorithms typically fail to capture. To address this, in human group we employed MD simulations to sample antibody–peptide conformations in addition to AF2/AF3 sampling, followed by AlphaFold2 refinement, to better accommodate the flexible nature of the peptide. H1223 is a representative case where the physics‐driven approach was particularly effective. Our method generated a structure with a DockQ score of 0.716 as the top‐ranked model, whereas the best AlphaFold‐Multimer (AF3) prediction we obtained prior to submission had a DockQ score of only 0.078.

### 3.3 Protein–Ligand Pose Prediction

**FIGURE 4:**
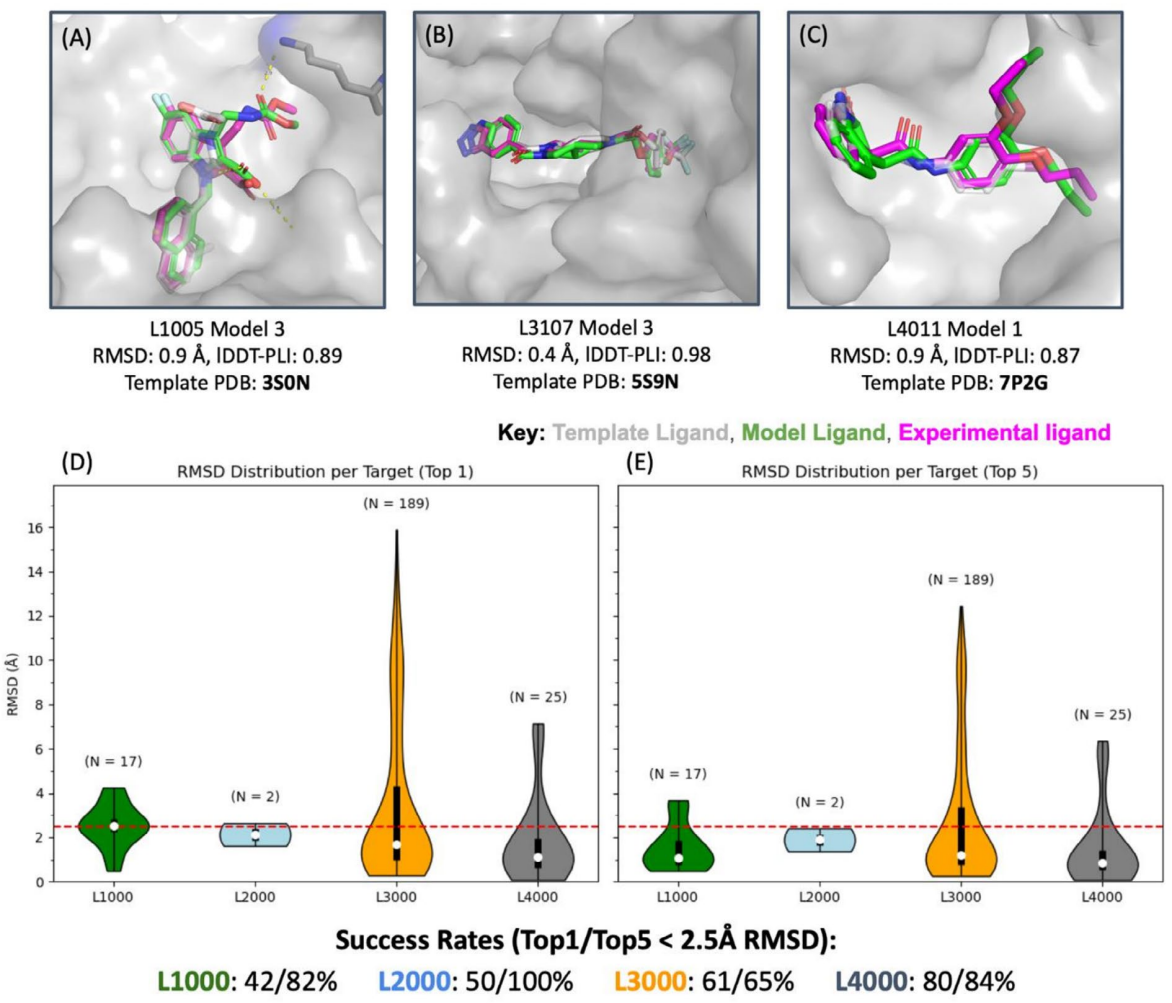
Top: Sample of accurate models obtained for ligands that bind to (A) Chymase, (B) Autotaxin, and (C) MPro. Bottom: Ligand RMSD distribution and success rates for the most accurate model in the top (D) 1 and (E) top 5 submissions, split by protein target. RMSD distributions for target ligands bound to Chymase (L1000) is displayed in green, Cathepsin G (L2000) is displayed in light blue, Autotaxin (L3000) is displayed in orange, and MPro (L4000) is displayed in gray. The number of ligands bound to each target protein is annotated above each respective violin plot. The dashed red line indicates an RMSD cutoff of 2.5 Å for a successful prediction.

In the CASP16 ligand docking challenge (233 complexes), our template‐based approach proved highly successful. We focus our analysis on drug‐like ligand targets (excluding trivial cases of small solvent molecules bound to proteins). Using the lDDT‐PLI metric [37]—which ranges from 0 (*completely incorrect pose*) to 1 (*all interactions predicted correctly*)—our method attained a mean lDDT‐PLI of 0.69 across all ligand targets. In practical terms, this corresponds to a large fraction of the interatomic contacts being predicted correctly for most complexes (Figure 4A–C). Additional ligand models with a broader range of lDDT‐PLI values are shown as Figure S1.

Breaking down the results by protein target provides additional insights. The CASP16 set included four distinct proteins, and our accuracy varied depending on the availability of template data for each. Furthermore, it was evident that more accurate docked poses could be obtained when robust pharmacophoric information was available. For example, Autotaxin (L3000) had an abundance of homologous complexes in the PDB, so our predictions were typically strong. The majority of Autotaxin ligands were modeled with near‐native accuracy, particularly when they shared substructures with motifs that bound within conserved pharmacophore regions. The advantage of using robust template information is illustrated particularly well when examining ligands that do not bind within the primary binding site of the receptor. When modeling the second ligand in target L3118, no robust substructure matches occurred with ligands in alternate binding pockets (Figure S2A). As a result, the ligand within the active site was modeled with a high degree of accuracy (RMSD = 1.0 Å), whereas the binding mode of the model in a proximal pocket deviated significantly from the crystallized structure (RMSD = 12.1 Å). Similarly, we could infer that target L3109 would bind within the same pocket as the crystallized sterol (PDB: 8C3O); however, a lack of robust structural overlap prevented our approach from producing an accurate model (Figure S2B). Across all pharmaceutical ligand targets, we find that accurate models are generally produced when templates are available. Among our top 1 models, predictions with a template available achieved a median RMSD of 1.51 Å, whereas those without a robust template had a median RMSD of 9.99 Å (Figure S3).

For SARS‐CoV‐2 Mpro (L4000), our method was the top performer by a wide margin, achieving an average lDDT‐PLI of 0.78, a significant improvement over the second‐best predictor group (LG262) who obtained an average lDDT‐PLI of 0.67 (see Figure S4). We attribute this to the wealth of coronavirus main protease complexes available as templates—the presence of many crystallographic ligands allowed us to construct a robust pharmacophore and SMARTS anchors.

Beyond overall scores, the CASP16 assessment also examined how often each method could place the ligand within a specific cutoff (for instance, RMSD ≤ 2.5 Å from the true pose, a common success criterion). We observed that our pipeline frequently succeeded in at least one of the top‐ranked submissions even if our top‐1 prediction was inaccurate (Figure 4D,E), which implies that the scoring protocol can be further improved. For example, in the Chymase set (L1000), a considerable portion of our best‐of‐five submissions for each ligand met the 2.5 Å RMSD threshold (Figure 4E), despite the top ranked pose not satisfying the cutoff (Figure 4D). In targets like Autotaxin and MPro, the difference between our top‐1 and top‐5 success rates was smaller, as our top prediction was often already correct. This indicates our ranking strategy was reasonably effective when a strong pharmacophore match made one pose stand out.

Qualitatively, we found that the explicit use of pharmacophore information was crucial to our success. In many instances, the initial placement of the ligand by our protocol—constrained by SMARTS to align key substructures—led to a ligand pose that “made sense” chemically, even before any scoring. For example, in Chymase and Cathepsin G complexes, our method correctly placed aromatic ring systems into a conserved hydrophobic pocket and oriented charged groups toward a conserved polar patch (Figure 4A). These choices were driven by recognizing common motifs from template inhibitors (like a benzene sulfonamide moiety that nearly all potent Chymase inhibitors share) and explicitly encoding that in the docking constraint. In cases where our top prediction was wrong, retrospective analysis often revealed that no template with a matching substructure had been available, or the protocol had anchored on a less relevant fragment, leading to an incorrect pose. Fortunately, such cases were relatively few in the CASP16 set. More commonly, if our pose was wrong, it was a scenario where multiple plausible poses existed (e.g., the ligand had symmetry or multiple rings that could flip), and our scoring did not sufficiently distinguish the correct one.

The key advantages of our method were its ability to incorporate known interaction patterns to improve the plausibility of predicted poses and its efficiency—by narrowing the search to relevant conformational space. The main limitations were the need for sufficient template data.

## 4 Discussion

In CASP16, we employed hybrid methodologies that integrate physics‐based modeling with machine learning approaches for the prediction of both protein–protein and protein–ligand interactions, and these strategies demonstrated competitive performance. In the case of protein–protein docking, the methodology necessitates a substantial degree of sampling. This observation naturally raises several important questions. From a theoretical perspective, one may ask what the fundamental limits of the approach are—specifically, whether the cases where our prediction was inaccurate could be recovered through more extensive physics and ML sampling. From an engineering perspective, the central issue is whether the approach can be rendered more computationally efficient, thereby enabling its effective deployment in large‐scale applications. Addressing both dimensions will require systematic benchmarking and detailed ablation studies, which we plan to undertake in future work.

For protein–ligand docking, the availability of structural templates emerged as a distinct double‐edged sword. In instances where suitable templates were abundant (e.g., in the cases of MPro and Autotaxin), our method performed exceptionally well. However, in situations where templates were scarce or where the ligand chemistry was particularly novel (such as with certain Chymase ligands), even our advanced template‐search strategies had limited structural information to exploit. These challenging cases underscore the necessity of further methodological development to better accommodate scenarios characterized by sparse template availability. Plate search had less to latch onto.

## 5 Conclusion

The CASP16 results of our group demonstrated that a judicious combination of physics‐based methods and machine learning can achieve state‐of‐the‐art performance in both protein–protein and protein–ligand structure prediction. For protein–protein docking, our pipeline leveraged AlphaFold2's exceptional accuracy for monomer structure and interface refinement, but crucially augmented it with a global docking search to escape local optima. For protein–ligand docking, we introduced a novel template‐based strategy that goes beyond naive substructure matching. This approach integrates machine learning‐based rescoring to improve pose selection. As a result, we correctly predict ligand poses in the majority of cases, even outperforming advanced AI methods, by capitalizing on prior structural knowledge embedded in the PDB. Although our methods achieved remarkable success for some of the CASP16 targets, they also pinpoint areas for future work, such as improving automated pose ranking and extending our template‐matching capabilities to cover more chemical space. In essence, the present paper suggests that hybrid methodologies that blend physical modeling and machine learning can improve structure prediction results. We anticipate that the community will further explore such integrative approaches, ultimately enabling researchers to confidently model molecular interactions at a speed and accuracy that rival experimental methods, thus accelerating drug discovery and our understanding of cellular machinery.

## Author Contributions

**Ryota Ashizawa:** conceptualization, methodology, software, writing – review and editing. **Sergei Kotelnikov:** conceptualization, methodology, software, writing – review and editing. **Omeir Khan:** conceptualization, methodology, writing – review and editing. **Stan Xiaogang Li:** methodology, investigation, writing – review and editing. **Ernest Glukhov:** methodology, investigation. **Xin Cao:** methodology, investigation. **Maria Lazou:** methodology, investigation. **Ayse Bekar‐Cesaretli:** methodology, investigation. **Derara Hailegeorgis:** methodology, investigation. **Veranika Averkava:** methodology, investigation. **Yimin Zhu:** methodology, investigation. **George Jones:** methodology, investigation. **Hao Yu:** methodology, investigation. **Dmytro Kalitin:** methodology, investigation. **Darya Stepanenko:** methodology, investigation. **Kushal Koirala:** methodology, investigation. **Taras Patsahan:** methodology. **Dmitri Beglov:** methodology, investigation. **Mark Lukin:** methodology, investigation. **Diane Joseph‐McCarthy:** methodology, investigation. **Carlos Simmerling:** methodology, investigation. **Alexander Tropsha:** methodology, investigation. **Evangelos Coutsias:** methodology, investigation. **Ken A. Dill:** methodology, conceptualization. **Dzmitry Padhorny:** conceptualization, methodology, software. **Sandor Vajda:** conceptualization, methodology, writing – review and editing. **Dima Kozakov:** conceptualization, methodology, software, writing – original draft, writing – review and editing.

## Conflicts of Interest

The PIPER docking program, which is the base of ClusPro, has been licensed by Boston University to Acpharis Inc. Acpharis, in turn, offers commercial sublicenses of PIPER. D.K. and S.V. consult for Acpharis and own stock in the company, and D.B. is the acting CEO of the company. However, the PIPER software and the ClusPro server are free for non‐commercial use.

## Supporting information


**Data S1:** prot70066‐sup‐0001‐Supinfo.pdf.

## Data Availability

Data are derived from public domain resources or are available by request.
