# Supplementary material for: Modeling Protein–Protein and Protein–Ligand Interactions by the ClusPro Team in CASP16
Source: Proteins. 2025 Oct 20;94(1):183–91. doi: 10.1002/prot.70066 (PMC12750026; doi:10.1002/prot.70066)
Supplement: Supplementary file 1 — Data S1: prot70066‐sup‐0001‐Supinfo.pdf. [file PROT-94-183-s001.pdf]

Supplementary Information

Modeling Protein-Protein and Protein-Ligand interactions by the ClusPro team in CASP16

Ryota Ashizawa[*1 2 3 4], Sergei Kotelnikov[*1 2 4], Omeir Khan[*5], Stan Xiaogang Li[1 2 3 4], Ernest Glukhov[1 2 3 4], Xin Cao[1 2 6], Maria Lazou[7], Ayse Bekar-Cesaretli[5], Derara Hailegeorgis[1 2 3 4], Veranika Averkava[1 2 3 4], Yimin Zhu[1 2 3 4], George Jones[1 2], Hao Yu[8], Dmytro Kalitin[1 2 4], Darya Stepanenko[1 2 4], Kushal Koraila[9], Taras Patsahan[10], Dmitri Beglov[7], Mark Lukin[11], Diane Joseph-McCarthy[5 7], Carlos Simmerling[2 12], Alexander Tropsha[9], Evangelos Coutsias[1 2], Ken A. Dill[2 12 13], Dzmitry Padhorny[†1 2 3 4], Sandor Vajda[†5 7], Dima Kozakov[†1 2 3 4 14]

1 Department of Applied Mathematics and Statistics, Stony Brook University, Stony Brook, NY, USA

2 Laufer Center for Physical and Quantitative Biology, Stony Brook University, Stony Brook, NY, USA

3 Oden Institute for Computational Engineering and Sciences, The University of Texas at Austin, Austin, TX, USA

4 Institute for Advanced Computational Science, Stony Brook University, Stony Brook, NY, USA

5 Department of Chemistry, Boston University, Boston, MA, USA

6 Simons Center for Computational Physical Chemistry, New York University, NY, USA

7 Department of Biomedical Engineering, Boston University, Boston, MA, USA

8 Department of Electrical & Computer Engineering, Boston University, Boston, MA, USA

9 Division of Chemical Biology and Medicinal Chemistry, UNC Eshelman School of Pharmacy, University of North Carolina at Chapel Hill, Chapel Hill, NC, USA

10 Institute of Applied Mathematics and Fundamental Sciences, Lviv Polytechnic National University, Lviv, Ukraine

11 Department of Pharmacological Sciences, Stony Brook University, Stony Brook, NY, USA

12 Department of Chemistry, Stony Brook University, Stony Brook, NY, USA

13 Department of Physics and Astronomy, Stony Brook University, Stony Brook, NY, USA

14 Department of Molecular Biosciences, The University of Texas at Austin, Austin, TX, USA

* These authors contributed equally to this work.

† Corresponding authors: dzmitry.padhorny@stonybrook.edu, vajda@bu.edu, midas@laufercenter.org

Supplementary Figures

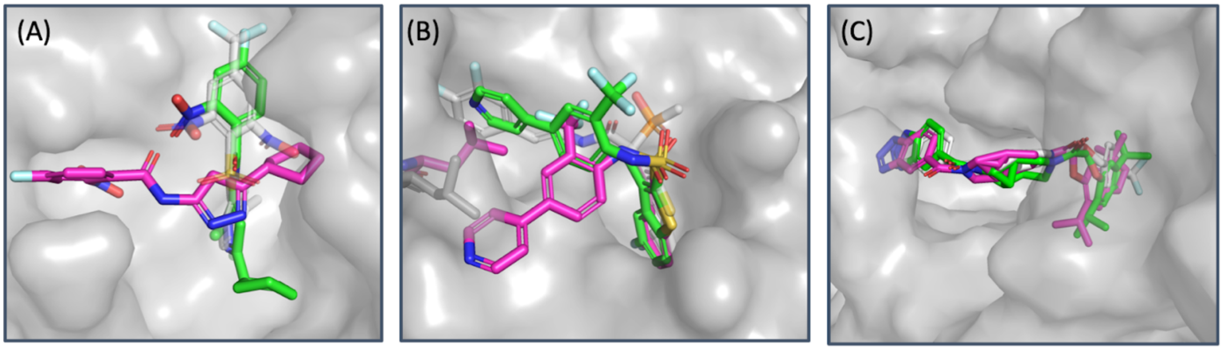

L4002 Model 1
RMSD: 7.1 Å , lDDT-PLI: 0.20
Template PDB: **2GZ7**

L1003 Model 3
RMSD: 3.6 Å , lDDT-PLI: 0.60
Template PDB: **3N7O**

L3103 Model 1
RMSD: 1.1 Å, lDDT-PLI: 0.78
Template PDB: **5S9N**

Key: Template Ligand, Model Ligand, Experimental ligand

Figure S1. Illustrative examples that demonstrate how lDDT-PLI changes with model quality for ligand targets (A) L4002, (B) L1003, and (C) L3103.

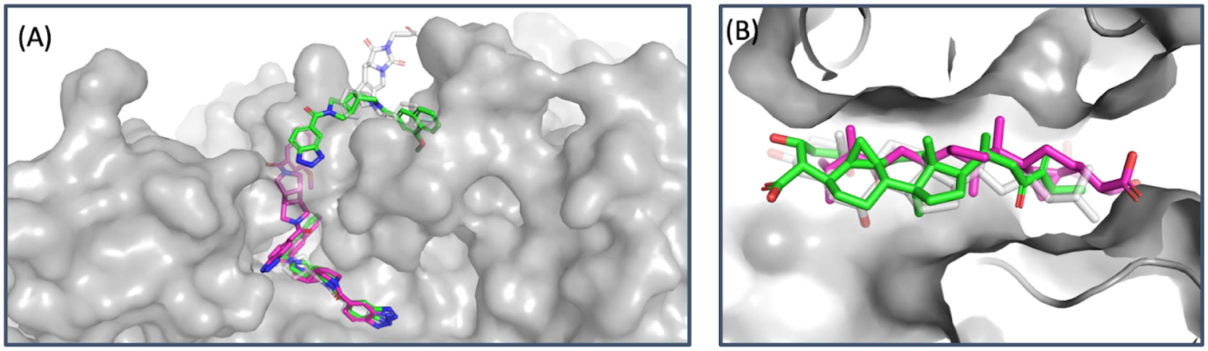

L3118 Ligand 1, Model 1
RMSD: 1.0 Å
Template PDB: **5S9M**

L3118 Ligand 2, Model 1
RMSD: 12.1 Å
Template PDB: 4ZG9

L3109 Model 1
RMSD: 11.0 Å
Template PDB: **8C3O**

Key: Template Ligand, Model Ligand, Experimental ligand

Figure S2. Submitted models for autotaxin targets where no robust template information was available. (A) Submitted binding modes for both ligands in target L3118. Ligand 1 denotes the copy of the ligand which binds within the primary binding site, and Ligand 2 denotes the copy of the ligand that binds within an alternate site where less templates are available. (B) Predicted binding mode for sterol target L3019.

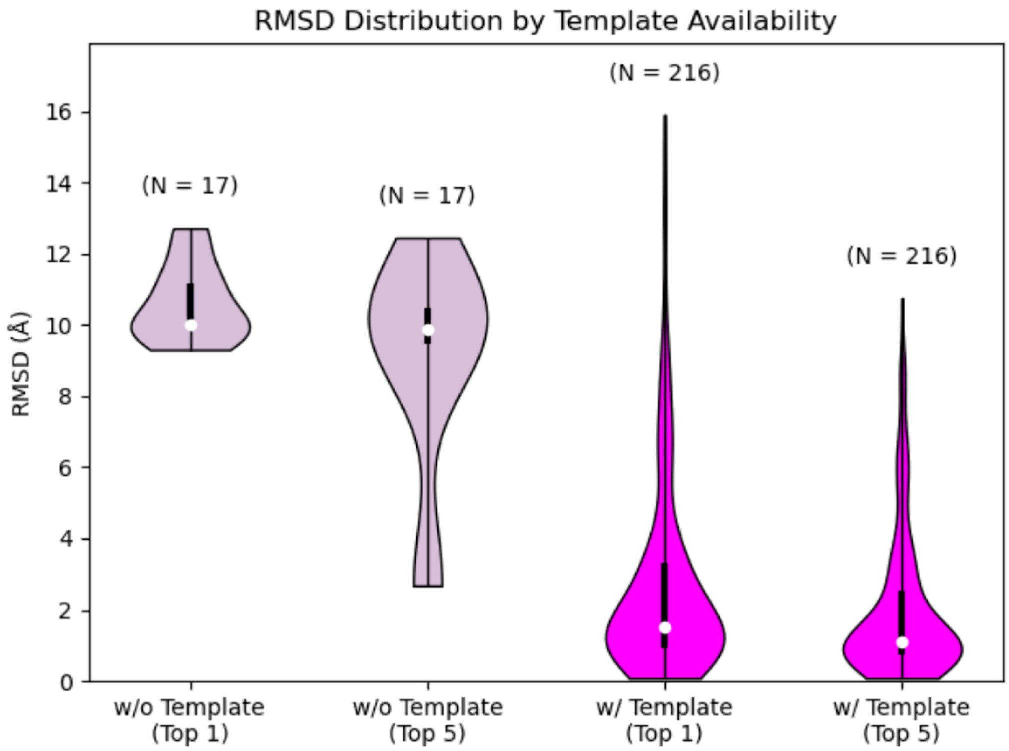

Figure S3. RMSD distributions for CASP ligand targets without (light pink) and with (fuchsia) template ligands available in the PDB. The distributions of the best RMSD model in the top 1 and top 5 ranked models is displayed. Medians are shown as a white dot, and interquartile ranges are shown as thick black bars.

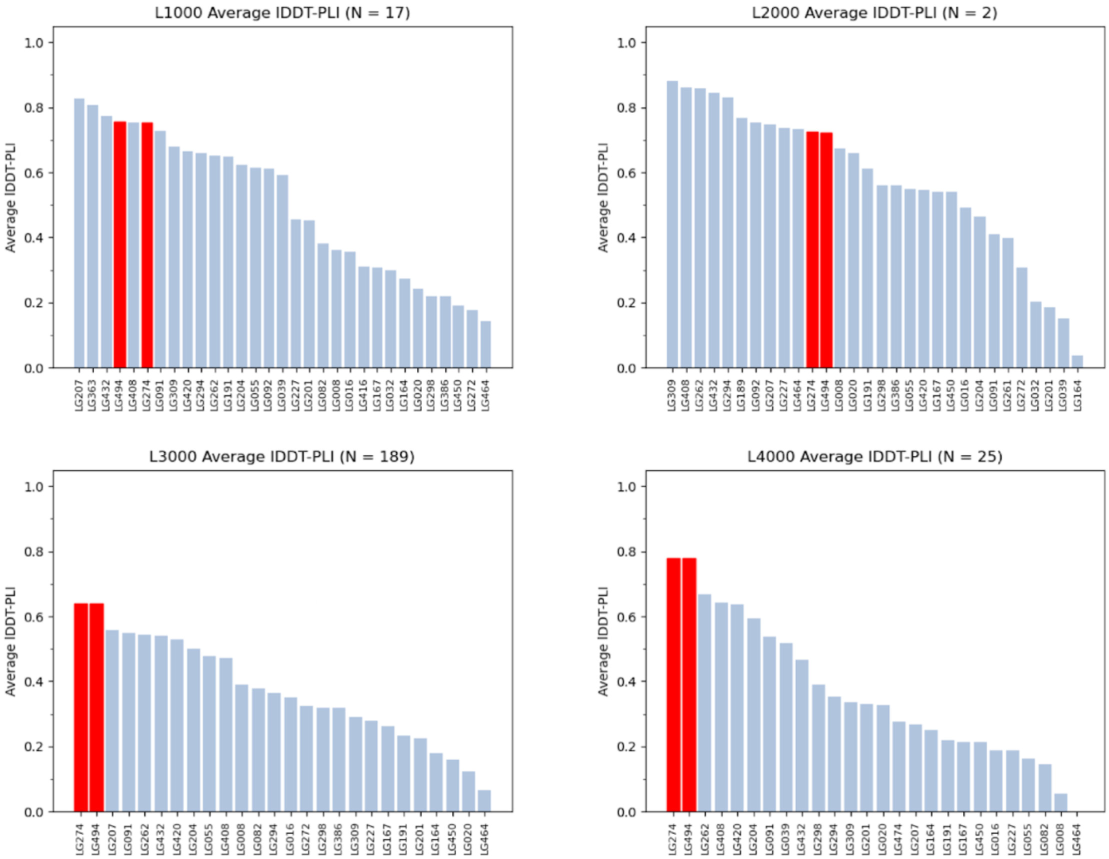

Figure S4. Average lDDT-PLI values for the top 1 model submissions for each predictor group, split by protein target. L4000 is the SARS-CoV-2 Mpro target. Results for our group (LG274 and LG494) are highlighted in red.
